# A Determinants-of-Fertility Ontology for Detecting Future Signals of Fertility Issues From Social Media Data: Development of an Ontology

**DOI:** 10.2196/25028

**Published:** 2021-06-14

**Authors:** Ji-Hyun Lee, Hyeoun-Ae Park, Tae-Min Song

**Affiliations:** 1 Nurse's Office Yeongdeok High School Gyeonggi-do Republic of Korea; 2 College of Nursing and Research Institute of Nursing Science Seoul National University Seoul Republic of Korea; 3 Department of Health Management Samyook University Seoul Republic of Korea

**Keywords:** ontology, fertility, public policy, South Korea, social media, future, infodemiology, infoveillance

## Abstract

**Background:**

South Korea has the lowest fertility rate in the world despite considerable governmental efforts to boost it. Increasing the fertility rate and achieving the desired outcomes of any implemented policies requires reliable data on the ongoing trends in fertility and preparations for the future based on these trends.

**Objective:**

The aims of this study were to (1) develop a determinants-of-fertility ontology with terminology for collecting and analyzing social media data; (2) determine the description logics, content coverage, and structural and representational layers of the ontology; and (3) use the ontology to detect future signals of fertility issues.

**Methods:**

An ontology was developed using the Ontology Development 101 methodology. The domain and scope of the ontology were defined by compiling a list of competency questions. The terms were collected from Korean government reports, Korea’s Basic Plan for Low Fertility and Aging Society, a national survey about marriage and childbirth, and social media postings on fertility issues. The classes and their hierarchy were defined using a top-down approach based on an ecological model. The internal structure of classes was defined using the entity-attribute-value model. The description logics of the ontology were evaluated using Protégé (version 5.5.0), and the content coverage was evaluated by comparing concepts extracted from social media posts with the list of ontology classes. The structural and representational layers of the ontology were evaluated by experts. Social media data were collected from 183 online channels between January 1, 2011, and June 30, 2015. To detect future signals of fertility issues, 2 classes of the ontology, the socioeconomic and cultural environment, and public policy, were identified as keywords. A keyword issue map was constructed, and the defined keywords were mapped to identify future signals. R software (version 3.5.2) was used to mine for future signals.

**Results:**

A determinants-of-fertility ontology comprised 236 classes and terminology comprised 1464 synonyms of the 236 classes. Concept classes in the ontology were found to be coherently and consistently defined. The ontology included more than 90% of the concepts that appeared in social media posts on fertility policies. Average scores for all of the criteria for structural and representations layers exceeded 4 on a 5-point scale. Violence and abuse (socioeconomic and cultural factor) and flexible working arrangement (fertility policy) were weak signals, suggesting that they could increase rapidly in the future.

**Conclusions:**

The determinants-of-fertility ontology developed in this study can be used as a framework for collecting and analyzing social media data on fertility issues and detecting future signals of fertility issues. The future signals identified in this study will be useful for policy makers who are developing policy responses to low fertility.

## Introduction

South Korea has the lowest fertility rate in the world. According to the Organization for Economic Cooperation and Development (OECD), the total fertility rate (TFR) in South Korea peaked in 1970 at 4.53 and subsequently declined to 1.30 in 2001 [1]. According to Statistics Korea [2], in 2018 the TFR fell to 0.98, below the critical level of 1.

In an attempt to increase the TFR, in 2005 the Korean government enacted the Basic Law on Low Fertility and Aging Society, and the Ministry of Health and Welfare in collaboration with other government agencies established 5-year plans. The First Basic Plan for Low Fertility and Aging Society (2006-2010) was initiated to establish a foundation for the government from which to proactively respond to the low fertility and aging population. The second and third of these plans (2011-2020) were pursued with the aim of increasing the TFR and successfully responding to the increasingly aging society [3-5]. However, despite the efforts of the government over the past 15 years, the TFR in South Korea remains the lowest in the world. In order to achieve the desired policy outcome of increasing the fertility rate, the government needs to continuously identify current issues related to fertility as well as those that will arise in the future based on the detection of future signals [6].

Governments around the world are increasingly seeking ways to detect future signals of policy implications so they can respond to the various challenges that countries face in a timely and effectively manner [6]. Government foresight programs such as the UK National Horizon Scanning Centre [7] and the Finland national foresight system [8] are monitoring future signals for policy making. Future signals are signals that are not currently mainstream but are useful for predicting changes in the future. Ansoff [9] defined such signals as weak signals, referring to small opinions or symptoms with unusual patterns of future changes. Weak signals are signs that do not impact the present but can develop into strong signals and then subsequently into a trend or megatrend in the future. Thus, the issues that will be important in the future may be predicted by detecting weak signals [10].

One approach to predicting future signals is to harness intuitive judgment by experts; however, this is both time-consuming and costly [11,12]. Since the volume of textual data and influence of public opinion on social media are increasing both rapidly and continuously, there have been attempts to detect future signals using social media data [12] in various areas, including the use of solar cells [12], school bullying [13], and health and welfare policies [14]. Policy makers can apply similar approaches to social media data to detect future signals of fertility issues in order to gain valuable insights and make better policy strategies toward increasing the fertility rate in South Korea [14].

Social media data are written in various forms and are both unstructured and noisy [15]. Analyzing such unstructured data requires not only a structured framework expressing a systematic domain classification and terminology, but also knowledge of the semantic relationships between concepts [16]. A framework based on semantic analysis is required to extract information from social media data that will be valuable to government policy makers [17]. In this study we propose an ontology as a framework for the analysis of social media data.

An ontology expresses shared concepts and their relationships in a specific domain [18], and it can be used as a framework for analyzing unstructured social media data since it systematically expresses knowledge as a set of concepts in a domain and incorporates the semantics of those concepts [19-21]. An ontology that includes terminology with synonyms of the ontology class concepts was found to be useful for analyzing the language commonly used by the general public on social media [20,21]. However, an ontology with terminology representing the determinants of fertility has yet not been developed.

This study aimed to (1) develop an ontology with terminology for collecting and analyzing social media data on the determinants of fertility, (2) determine the description logics (DL), content coverage, and structural and representational layers of the ontology, and (3) use the ontology with terminology to detect future signals of fertility issues in social data posted in Korean.

## Methods

### Ontology and Terminology Development

An ontology for describing the determinants of fertility, called the determinants-of-fertility ontology, was developed based on the Ontology Development 101 methodology [22] in 5 steps, as described below.

#### Step 1. Determining the Domain and Scope of the Ontology

The aim of the determinants-of-fertility ontology developed in this study was to analyze social media data posted by consumers, not by health care professionals. Thus, we limited the scope of the ontology to the individual, social, economic, cultural, and policy factors of fertility in the domain of the consumer. The physiological, clinical, and therapeutic factors of fertility in the domain of health care professionals were excluded. The specific domain and scope of this ontology was determined by creating competency questions (CQs) that the ontology must be able to answer [23]. Since fertility is affected by multilevel factors [24], the domain and scope of the ontology were determined based on the ecological model [25]. CQs were extracted on the reproductive decisions of women from a report on low fertility in OECD countries [26] and from a research report on the causes of low fertility in South Korea [27], such as “What are the personal factors that influence a woman’s decision to have a child?” and “What are the Korean government’s policies for overcoming low fertility?” The CQs were also subsequently used for evaluating the ontology.

#### Step 2. Considering Reusing Existing Ontologies

We identified existing ontologies and conceptual frameworks representing fertility by searching PubMed, Google Scholar, and BioPortal [28] using the keywords “fertility,” “childbirth,” “low fertility,” “fertility ontology,” “childbirth ontology,” and “low-fertility ontology.” This search process identified an ontology representing genes associated with infertility, but this ontology was not appropriate for this study since it only included genetic factors related to infertility.

#### Step 3. Extracting Important Terms in the Ontology

We extracted terms from the literature that were consistent with the domain and scope of the ontology. The literature reviewed included reports on fertility, determinants of fertility, low fertility, and policy responses to low fertility published by the OECD [29], United Nations Population Division [30], and United Nations Population Fund [31], which are international organizations dealing with fertility issues jointly in countries around the world. We also searched the literature using the keywords “childbirth,” “fertility,” “determinants of fertility,” and “low fertility rate.” The reviewed literature included research papers on individual socioeconomic factors affecting low fertility and policies in countries experiencing low fertility problems such as South Korea, Japan, Singapore, Spain, Portugal, the United States, and France. Additional terms were extracted by reviewing social media posts and national surveys on fertility issues.

#### Step 4. Defining the Classes and Class Hierarchy

The classes of the ontology and their hierarchy were defined using a top-down approach. The superclasses of the ontology and their relationships were constructed by integrating an ecological model [25].

#### Step 5. Defining the Internal Structure of Classes

The internal structure of the ontology classes was defined by adding the properties of the classes, the value of the properties, and the value type using the entity-attribute-value (EAV) model. Entities refer to the concepts covered in the determinants of fertility, attributes are characteristics of entities, and value sets comprise the set of values that an attribute can have. Attributes and values were extracted from the questionnaires of the Korean National Survey on Dynamics of Marriage and Fertility [32] and the Korean Longitudinal Survey of Women and Families [33]. The ontology also included terminology with a list of synonyms for classes, attributes, and values. We formally represented the determinants-of-fertility ontology using open-source Protégé software (version 5.5.0).

### Ontology and Terminology Evaluation

The available methods for evaluating the quality of an ontology include those proposed by Brank et al [34], Obrst et al [35], and Vrandečić [36]. Brank et al [34] classified ontology evaluations into 4 categories: (1) approaches that compare the target ontology to a gold standard (gold standard), (2) approaches that use the target ontology in an application (application-based), (3) approaches that compare the target ontology with a source of data (data-driven), and (4) approaches that assess the target ontology by human (user-based). Since there is no gold standard available, the determinants-of-fertility ontology was evaluated using the remaining 3 approaches proposed by Brank et al [34] (ie, application-based, data-driven, and user-based evaluations). The ontology was revised based on the evaluation results.

#### Evaluating the DL of the Ontology

We tested the DL of the ontology by applying the ontology debugger Protégé plug-in. We also tested the DL using the DL-reasoner Protégé plug-in to determine whether the ontology generates the correct answers to the previously developed CQs. For example, the CQ “What are the personal factors that influence a women’s decision to have a child?” was converted to a DL query “IsIndividualOf some Determinants_of_fertility.” After entering this query into Protégé, we tested whether the answers to the CQ were correct. Since the determinant of the fertility class (domain) was related to the subclasses of individual (range) through the *hasIndividual* relationship, and subclasses (eg, sociodemographic data, reproductive health, and individual’s attitude) of the individual were related to the individual class through an *is-a* relationship, we could obtain the result of a DL query.

#### Evaluating the Content Coverage of the Ontology

The content coverage of the ontology was examined by comparing terms extracted from the bulletin board of the Korean Ministry of Health and Welfare with a list of classes and synonyms of the ontology. Both the general public and public servants are allowed to post their opinions or concerns on fertility issues and policies regarding low fertility on this bulletin board. In total, 1387 documents posted on the website by the general public and 63 posted by public servants were collected. Relevant terms in the documents were extracted using the Korean Natural Language Processing package in R software (version 3.2.1, R Foundation for Statistical Computing). Unique concepts were extracted based on the meaning of the terms and then mapped onto the ontology classes. The mapping results were reviewed by 3 experts in health informatics who had experience in ontology development [37]. Any new concepts that were identified were added to the ontology.

#### Evaluating the Structural and Representational Layers of the Ontology

The structural and representational layers of the ontology were evaluated by 3 experts in health informatics who had previous experience in ontology design and 2 experts in maternity nursing who had previous experience in ontology evaluation. The evaluation tool developed by Jung et al [38] was used; this tool was based on the criteria for the structural and representational layers of Kehagias et al [39]. The structural layer was evaluated using 7 items: size, hierarchy depth, hierarchy breadth, density, balance (equally developed), overall complexity, and connectivity between classes. The representational layer was evaluated using 10 items: the match between formal and cognitive semantics, consistency, clarity, explicitness, interpretability, accuracy, comprehensiveness, granularity, relevance, and description. Each item was scored on a 5-point scale ranging from 1 (strongly disagree) to 5 (strongly agree). The structural layer was evaluated with the entire ontology based on the hierarchy of classes and relationships between the classes, and the representational layer was evaluated with each of 6 superclasses based on the EAV model of each class.

### Applying the Ontology to Detect Future Signals

The ontology with terminology was used to detect future signals of fertility issues from social media data. Future signals were analyzed based on the text-mining–based weak-signal detection method of Yoon [12], as follows, using R software (version 3.5.2).

#### Step 1. Collecting Data

We collected posts on fertility issues written in Korean from the following 183 online channels between January 1, 2011, and June 30, 2015: 159 channels of online news, 17 message boards, 1 social networking service (Twitter), 4 internet blogs, and 2 online community services. “Low fertility” was used as a major search keyword, together with synonyms of “fertility rate decline,” “sharp decline in fertility rate,” “avoiding childbirth,” “no kids,” and “childless family.” Social media data were collected using the SK telecom’s big-data analytics platform [40]. The data were preprocessed by treating a single document as an analysis unit.

#### Step 2. Defining the Keywords

After extracting terms from each document, we identified the terms related to fertility issues such as socioeconomic and cultural factors and fertility policies. The future signals of fertility issues were detected using the keywords representing socioeconomic and cultural factors and fertility policies. The keywords that were semantically similar but expressed using different terms [12] were grouped into class concepts of the ontology using terminology linking terms to concepts. The top 17 most frequently encountered keywords were selected for future-signal analysis (Textbox 1), which excluded rarely used keywords that are likely to affect the average frequency and growth rate obtained in such analyses [13,41]. The document was then coded based on whether keyword was absent (=0) or present (=1) in order to check the document frequency (DF) for the occurrence of keywords.

**Selected keywords.**

Socioeconomic and cultural factors:

- Population aging
- Economic problems
- Nuclearization of the family
- Changing perspectives about marriage
- Conservative values
- Violence and abuse
- Employment problems
- Gender inequality

Fertility policies:

- Financial support for childbirth
- Child-safety protection system
- Infrastructure for childcare support
- Maternity-leave system
- Policy public relations
- Financial support for employment security
- Flexible working arrangement
- Family-friendly work environment
- Smart work center

#### Step 3. Constructing Keyword Portfolio Maps

Future signals (also defined as weak signals) show abnormal patterns due to current oddities [10]. Future signals can be detected by constructing keyword portfolio maps using the frequency information and by applying a time-weighted approach to focus on recent abnormalities [12]. We constructed a type of keyword portfolio map called a keyword issue map (KIM) using the growth rate of the degree of diffusion (DoD) for the DF of keywords. The KIM shows the extent to which future-signal topics are diffused. The DF represents how common the term is in the collected documents. Since terms that occur frequently within collected documents are more important, the DF is directly related to future signals and can be calculated as:

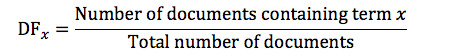



The DoD is the growth rate of the term occurrence expressed as a time-weighted coefficient and is also important for detecting future signals. The DoD represents how the diffusion of a term across different documents varies over time. Since the recent appearance of a term is more important than its past appearance, the DoD puts more weight on recent occurrences:

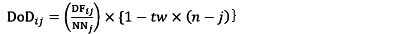



where DF*_ij_* is the DF of keyword *i* during period *j*, NN*_j_* is the total number of documents identified for period *j*, *n* is the number of periods, and *tw* is a time weight (previous studies have used *tw* = 0.05 [12,41]).

The KIM was generated by plotting the average DF on the x-axis and the average growth rate of the DoD on the y-axis. The quadrants of the plot were divided by the medians of the respective values, and so each quadrant of the KIM represented different information about present and future keywords.

#### Step 4. Identifying Weak-Signal Topics

Future signals were identified according to where keywords were located in the quadrants of the KIM. Keywords in the first quadrant, which represent strong signals, have a trend toward a high average DF and a high average DoD growth rate. Keywords in the second quadrant, which represent weak signals, have a low average DF but a high average DoD growth rate, and so they may increase rapidly in the future. Keywords in the third quadrant, which represent latent signals, have a low average DF and a low average DoD growth rate and are not yet significantly noticeable. Keywords in the fourth quadrant, which represent not-strong-but-well-known signals, have a high average DF but a low average DoD growth rate, and so currently exhibit a slow growth rate.

## Results

### Ontology and Terminology Development

A list of 10 CQs was compiled (Textbox 2) that reflect different levels of factors affecting fertility using an ecological model. The domain and scope of the ontology were determined based on CQs for the determinants of fertility related to the individual, family, workplace, childcare and educational environment, socioeconomic and cultural environment, and public policy. Childbirth before marriage is very uncommon in South Korea, and so delayed marriage is an important factor affecting childbirth decision-making [3,4,27,42,43]. Therefore, the scope of fertility determinants included factors related to marriage delay and childbirth decision-making.

**Competency questions.**

1. What are the personal factors that influence a woman’s decision to have a child?
2. What are the family factors that influence the decision to have a child?
3. What are the childcare factors that influence the decision to have a child?
4. What are the educational factors that influence the decision to have a child?
5. What are the workplace factors that influence the decision to have a child?
6. What are the sociocultural factors that influence the decision to have a child?
7. What are the economic factors that influence the decision to have a child?
8. What is the Korean government’s policy for overcoming low fertility?
9. What are the policy tasks for addressing low fertility in South Korea?
10. What are the policy targets for low fertility in South Korea?

In total, 1659 terms covering the domain and scope of the ontology were collected, and 236 unique class concepts were extracted from these terms. We defined hierarchical and attribute relationships of the classes based on the ecological model. The determinants of fertility were organized into the following levels: individual, family, workplace, childcare and educational environment, socioeconomic and cultural environment, and public policy. These 6 levels of the ontology were defined by adding not only the workplace, but also childcare and educational environment to institutional factors, which constitute the third level of the ecological model. Due to the increasing participation of women in the labor market, the workplace and childcare and educational environment are important factors influencing decisions about childbirth among women who are working [3,4,27,32,42,43]. Employed women in South Korea experience difficulties combining working with childbirth and childcare and are often faced with choosing between giving up their jobs to give birth and look after their child or continuing to work, thereby forsaking their desire to give birth and look after a child [32,42,43]. Therefore, we viewed the workplace and the childcare and educational environment as important social institutional factors and determinants of fertility.

**Figure 1:**
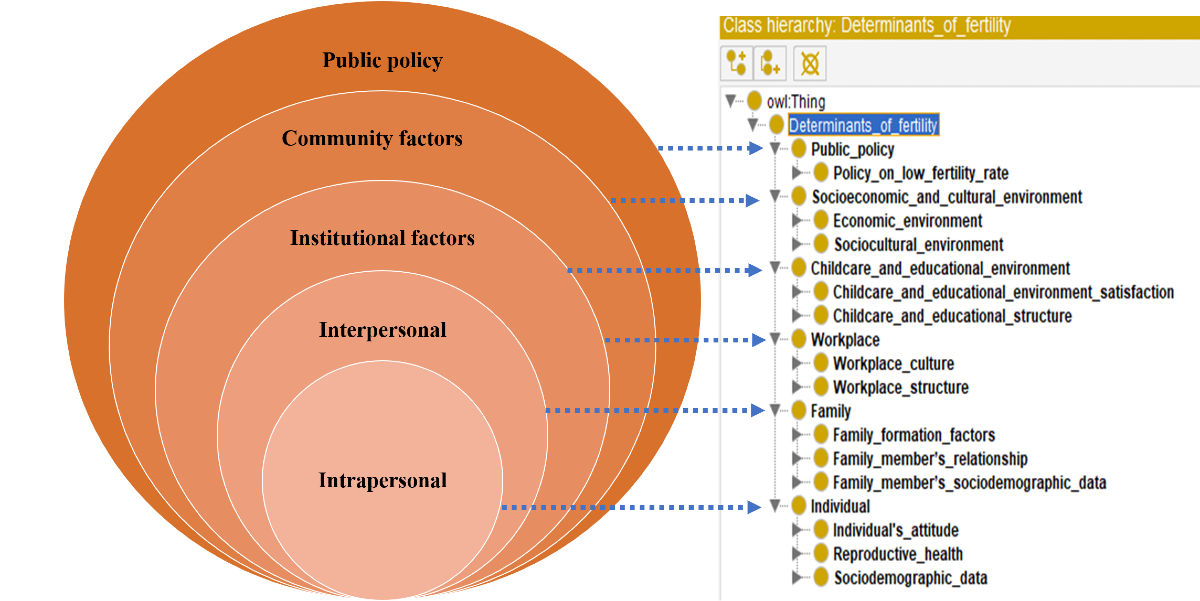
The determinants-of-fertility ontology based on an ecological model.

Figure 1 shows the determinants-of-fertility ontology with classes up to the second level. The ontology consists of 6 superclasses: individual, family, workplace, childcare and educational environment, socioeconomic and cultural environment, and public policy. The individual superclass has the subclasses of women’s sociodemographic data, which include age, education, employment, and religion; reproductive health, which includes sexual behavior, contraceptive use, and childbirth; and the individual’s attitude, which includes their attitude toward children and marriage. The family superclass has the subclasses of the family’s sociodemographic data, which include family size, family income, family expenditure, sex and age of children, number of children, spouse’s age, and spouse’s income; the family member’s relationship, which includes gender equality, couple’s intimacy, and family’s life satisfaction; and family-formation factors, which include marriage cost. The workplace superclass has the subclasses of workplace structure, which includes workplace type, working hours, and employment insurance, and workplace culture, which includes parental leave availability. The childcare and educational environment superclass has the subclasses of the childcare and educational structure, which includes type, cost, service, and human resources, and childcare and educational environment satisfaction, which includes the belief of the quality. The socioeconomic and cultural environment superclass has subclasses of the sociocultural environment, which includes social value, mass media, and social change, and the economic environment, which includes economic growth. Finally, the public policy superclass has the subclass of policy on low fertility, which includes the area, legal basis, and tasks. These classes had 3 or 4 levels of hierarchy with 230 classes and 41 relationships.

We developed EAV models for the 139 lowest level class concepts. For example, *contraceptive use* had attributes of *hasType* and *hasLengthOfUse*, where the values of the *hasType* attribute were *condoms*, *contraceptive injection*, *contraceptive patch*, *pill*, *intrauterine device*, *diaphragm*, and *sterilization* and *hasLengthOfUse* had the value of *number of months*. We also developed a terminology linking synonyms for classes, attributes, and values: 90 synonyms for 236 classes, 9 synonyms for 54 attributes, and 501 synonyms for 772 values.

### Ontology and Terminology Evaluation

#### Evaluating the DL of the Ontology

The Protégé ontology debugger program revealed that concept classes in the ontology were coherently and consistently defined. The DL reasoner showed that the ontology correctly answered all 10 CQs.

#### Evaluating the Content Coverage of the Ontology

The content coverage of the ontology is presented in Table 1. In total, 751 terms were extracted from the narratives posted by the general public and public servants. We extracted 532 unique concepts from the terms, of which 494 (92.9%) were included in the ontology. Examples of new concepts are test tube (in vitro fertilization), health insurance, and administrative division, and we added such new concepts to the ontology. The ontology was revised by adding 18 synonyms for classes, 17 value concepts, and 3 synonyms for values. The finalized version of the determinants-of-fertility ontology comprised 6 superclasses, 108 synonyms for 230 classes, 9 synonyms for 54 attributes, and 504 synonyms for 789 values.

**Table 1:** Results for the content coverage of the ontology.

Category	General public, n (%)	Public servants, n (%)	Total, n (%)
Existing concepts	416 (92.0)	93 (97.9)	494 (92.9)
New concepts	36 (8.0)	2 (2.1)	38 (7.1)
Total	452 (100)	95 (100)	532 (100)

#### Evaluating the Structural and Representational Layers of the Ontology

Average scores for all of the criteria for structural and representations layers exceeded 4 on a 5-point scale. The experts rated the hierarchy breadth, density, overall complexity, and connectivity criteria as strongly agree (score 5). The criterion with the lowest score was accuracy of the representation layers, with a score of 4.33 (Table 2).

**Table 2:** Results for the structural and representational layers of the ontology.

Criteria		Average score (range)
**Structural layer**		
	Size	4.80 (4-5)
	Hierarchy depth	4.60 (4-5)
	Hierarchy breadth	5.00 (5-5)
	Density	5.00 (5-5)
	Balance	4.60 (4-5)
	Overall complexity	5.00 (5-5)
	Connectivity	5.00 (5-5)
**Representational layer**		
	Match between formal and cognitive semantics	4.73 (4-5)
	Consistency	4.50 (4-5)
	Clarity	4.87 (4-5)
	Explicitness	4.60 (3-5)
	Interpretability	4.67 (4-5)
	Accuracy	4.33 (4-5)
	Comprehensiveness	4.77 (4-5)
	Granularity	4.47 (3-5)
	Relevance	4.83 (4-5)
	Description	4.83 (4-5)

### Applying the Ontology to Detect Future Signals

Table 3 lists the results for the computed DoD for each keyword for the socioeconomic and cultural factors and fertility policies. *Violence and abuse* (socioeconomic and cultural factor) and *flexible working arrangement* (fertility policy) had a low DF and high DoD growth rates. *Economic problems*, *population aging*, and *nuclearization of the family* (socioeconomic and cultural factors) and *child-safety protection system* (fertility policy) had a high DF and high DoD growth rates.

**Table 3:** Degree of diffusion (DoD), average DoD growth rate, and average document frequency for fertility issues.

Category and keyword			DoD^a^									Average DoD growth rate		Average DF^b^
			2011		2012	2013		2014		2015				
**Socioeconomic and cultural factors**														
	Population aging	7463		8912		8002	4499		4503		0.088		6676	
	Economic problems	1637		2054		2523	1471		1503		0.214		1838	
	Nuclearization of the family	1178		1288		1229	667		628		0.054		998	
	Changing perspectives about marriage	1046		1528		1116	596		484		0.034		954	
	Conservative values	1150		1195		1139	576		565		0.036		925	
	Violence and abuse	685		800		726	461		527		0.158		640	
	Employment problems	515		510		436	306		208		–0.008		395	
	Gender inequality	286		298		319	190		133		0.027		245	
**Fertility policies**														
	Financial support for childbirth	3061		3145		2548	1573		1250		–0.015		2315	
	Child-safety protection system	1757		1632		1974	1241		1292		0.156		1579	
	Infrastructure for childcare support	1853		2209		1310	829		579		–0.061		1356	
	Maternity-leave system	1067		995		798	828		383		0.044		814	
	Policy public relations	878		883		894	648		361		0.015		733	
	Financial support for employment security	392		341		345	233		196		0.045		301	
	Flexible working arrangement	330		264		354	287		180		0.120		283	
	Family-friendly work environment	161		130		77	49		35		–0.146		90	
	Smart work center	131		114		90	50		27		–0.161		82	

^a^DoD: degree of diffusion.

^b^DF: document frequency.

**Figure 2:**
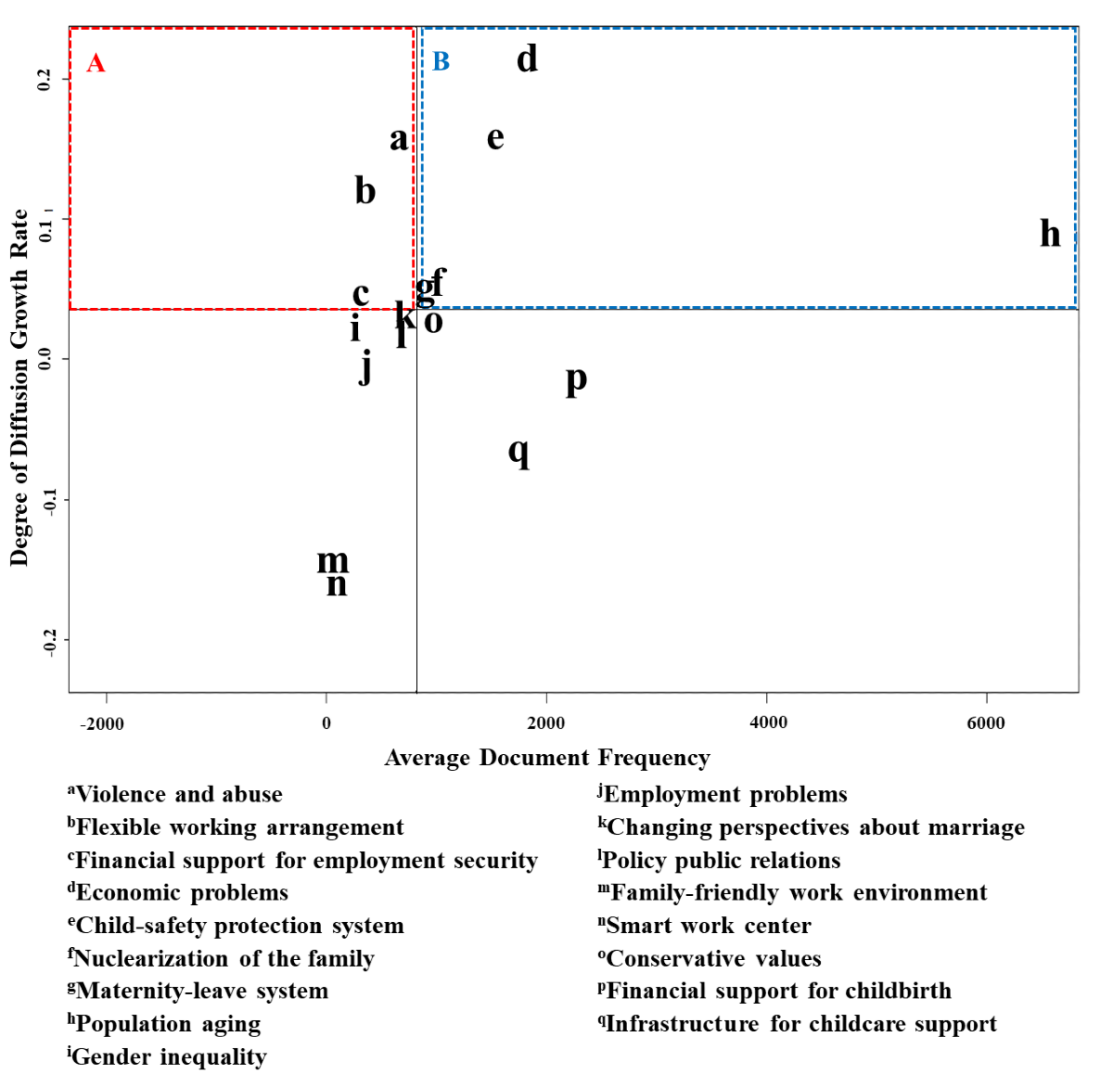
Future signal classification using the keyword issue map of fertility issues. Red rectangle (area A) indicates weak signals and blue rectangle (area B) indicates strong signals.

Figure 2 shows the KIM. The weak signals are marked with red rectangle (area A) in the second quadrant of the KIM. The strong signals are marked with blue rectangle (area B) in the first quadrant of the KIM. Table 4 presents the signal classification of keywords for socioeconomic and cultural factors and fertility policies in the KIM. The keywords classified as weak signals were *violence and abuse* (socioeconomic and cultural factor) and *flexible working arrangement* and *financial support for employment security* (fertility policies). The keywords classified as strong signals were *economic problems*, *nuclearization of the family*, and *population aging* (socioeconomic and cultural factors), and *child-safety protection system* and *maternity-leave system* (fertility policies). The keywords classified as latent signals were *gender inequality*, *employment problems*, and *changing perspectives about marriage* (socioeconomic and cultural factors), and *policy public relations*, *family-friendly work environment*, and *smart work center* (fertility policies). The keywords classified as not-strong-but-well-known signals were *conservative values* (socioeconomic and cultural factor) and *financial support for childbirth* and *infrastructure for childcare support* (fertility policies).

**Table 4:** Future signal classification of fertility-issues keywords.

Category and weak signals		Strong signals	Latent signals	Not-strong-but-well-known signals
**Socioeconomic and cultural factors**				
	Violence and abuse	Economic problems	Gender inequality	Conservative values
	—^a^	Nuclearization of the family	Employment problems	—
	—	Population aging	Changing perspectives about marriage	—
**Fertility policies**				
	Flexible working arrangement	Child-safety protection system	Policy public relations	Financial support for childbirth
	Financial support for employment security	Maternity-leave system	Family-friendly work environment	Infrastructure for childcare support
	—	—	Smart work center	—

^a^Not applicable.

## Discussion

### Principal Results

We have developed a determinants-of-fertility ontology as a framework for collecting and analyzing social media data. The ontology was evaluated in terms of the DL, content coverage, and structural and representational layers. We applied the ontology with terminology to detect future signals of fertility issues from social media data.

The developed determinants-of-fertility ontology has 6 main characteristics. First, it is the first ontology to describe the multilevel factors that affect fertility. Various factors and the complex interactions between them determine fertility [24,26,27], and expressing the fertility determinants requires an integrated view of these factors. We therefore developed the ontology by classifying the various environmental factors related to the individuals and in terms of family, childcare, workplace, and community levels from an ecological perspective.

Second, this ontology contains factors related to fertility issues that are unique to South Korea. In most cases, childbirth does not occur until after marriage in South Korea, and delaying marriage is an important factor affecting the decision to have a child [27,42,43]. Therefore, the scope of the ontology spanned individual and social factors that influence not only delayed marriage but also the decision to have children after marriage. For example, the ontology includes concepts of attitude toward marriage in the individual superclass, matters related to family formation in the family superclass, and support for starting a family in the public policy superclass.

Third, the developed ontology includes terminology with synonyms for classes such as consumer terms and abbreviations, which makes it suitable for analyzing social media data. For example, regarding *financial support for childbirth*, various terms such as *childbirth celebration money*, *childbirth grant*, *subsidy*, *baby bonus*, and *childbirth incentive* are used on social media postings. Since the developed ontology includes these terms, it can be used to collect and analyze consumer terms in social media data.

Fourth, each class of this ontology was modeled using the EAV model and included the attributes of each class and the values of those attributes. Like previous research [20,21], value sets of attributes representing the level and status of an entity included terms describing the keyword in detail in social media data. This novel characteristic of our ontology renders it capable of advanced keyword extraction and suitable for analyzing social media data.

Fifth, we ensured quality of the ontology by using a variety of evaluation methods, including the application-based, data-driven, and user-based approaches proposed by Brank et al [34]. Application-based evaluation was performed by testing the ability of the ontology to answer the CQs that cover its domain and scope. The ontology provided correct answers to all of the CQs without any errors using DL. Data-driven evaluation was performed by testing the content coverage of the ontology whereby terms extracted from social media posts on fertility issues were compared with terms included in the ontology. Medical terms related to infertility and pregnancy that appeared on social media were not included in the ontology. As a result, medical terms on social media were added to the ontology. User-based evaluation was performed by asking the experts to rate the structural consistency, errors in class relationships, and representational quality of the ontology using the evaluation criteria of the structural and representational layers [39]. The experts rated all the evaluation criteria at between 3 and 5 on a 5-point scale.

Finally, the ontology with terminology developed in this study was used as a framework to detect future signals of fertility issues from social media data. The ontology allowed us to use social media data to identify the current trends and future changes in fertility issues related to the effective implementation of policies to increase the fertility rate. These trends were *economic problems*, *child-safety protection system*, *violence and abuse*, and *flexible working arrangement*.

*Economic problems* and *child-safety protection system* were strong signals of fertility issues. *Economic problems* were noted to be an important topic in a survey about public perceptions of the low fertility phenomenon [44]. In a study by the Korean Ministry of Health and Welfare involving 2000 adults that examined the perceptions of low fertility and population aging, the participants reported that the main causes of low fertility were economic problems such as the economic burden of child support and education costs (60.2%) and employment instability (23.9%) [44]. Therefore, there is a need for governments to provide continuous support measures to reduce economic difficulties in family formation, childbirth, and parenting.

A *child-safety protection system* needs to be implemented as another investment toward avoiding low fertility in future generations [45]. In order to protect the health and safety of children, the government needs to actively respond to risk factors that threaten child safety such as child abuse and school violence [3,4,45-47]. Policies on child safety have been one of the most important national issues since 2013. The Korean government that came into power in 2013 has focused on protecting vulnerable groups such as children, adolescents, and women by introducing policies to deter sexual abuse, school violence, and domestic violence [46,47]. Since the government promoted these policies, both the mainstream media and social media treated them as big issues [46,47]. However, only 13.1% of married couples reportedly felt that their children were growing up in a safe and healthy environment [48], and researchers have continued to point out that the weak child-protection system remains a problem in South Korea [45,48,49]. The government therefore needs to focus more on solving the limitations and problems associated with the *child-safety protection system*.

*Violence and abuse* and *flexible working arrangement* were weak signals that could develop into strong signals in the future. *Violence and abuse* were previously reported as important factors for low fertility, with experts identifying changes in the social environment as being important [45,49,50]. The proportion of children (younger than 18 years) in the total population has decreased from 19.6% in 2013 to 15% in 2019 due to the low fertility rate, while the reported number of child-abuse cases has increased from 10,943 in 2012 to 36,417 in 2018 [50]. South Korea is still a highly patriarchal and authoritarian society, and a child is viewed as a subject to be parented and disciplined rather than as one with its own rights [49,50]. Thus, it is necessary to change the perception of violence and abuse toward children as social problems rather than family problems. The government needs to continually encourage policies that not only improve the fertility rate but also improve the environment that currently threatens the safety and health of babies and children.

A *flexible working arrangement* is a policy that allows workers to balance work and life [4,51]. Both the OECD and the Korean Ministry of Health and Welfare promote this as an important policy for increasing the future fertility rate in South Korea [52]. Long working hours, long commuting hours, and the culture of socializing after work in South Korea make it difficult to maintain an optimal balance between work and family [52]. Therefore, solving the low fertility problem in this country will require the government to promote policies enabling workers to have flexible working hours.

### Limitations

The determinants-of-fertility ontology developed in this study comprehensively covers fertility issues relevant to the low fertility phenomenon in South Korea and will be useful for analyzing social media data. However, it is also subject to several limitations.

First, the direct and indirect effects of employment stability, job creation, housing supply, and public education on fertility [53] were not included in the public policy category of the ontology because the Korean government applies these policies separately from the policies for low fertility. A comprehensive approach covering relevant policies is needed to effectively address low fertility. Future fertility research should include policy strategies that address issues related to employment, job creation, housing supply, and public education.

Second, the synonyms of the ontology developed in this study may not include all the terms used by the general public on social media. Many of the terms used on social media are highly transient—rapidly appearing, spreading, and then disappearing [54,55]. The terms included in the ontology therefore need to be updated continuously based on those currently used by the general public.

Third, future signals of fertility issues were detected during the second phases of the policy on low fertility. Low fertility is a demographic issue that requires a long-term approach, and the policy responses of the government should be periodically reviewed and evaluated to ensure that the policies in place at a particular time point are consistent with any changes in the population and the socioeconomic and cultural environment [56]. This situation indicates the need to analyze social media data periodically using the ontology developed in this study to establish the policy direction for addressing the low fertility phenomenon by reflecting current and future trends on fertility issues as accurately and timely as possible.

Finally, only the KIM that uses the DF of keywords was used to detect future signals. Since future signals are generally subjective [10], even a careful analysis of future signals will not always yield accurate results [57]. Yoon [12] proposed a quantitative method using 2 types of keyword portfolio maps—a KIM using the DF and a keyword emergence map using the keyword frequency—to accurately detect future signals. In contrast, Lee and Park [57] proposed using both quantitative and qualitative methods to ensure the accurate detection of future signals. Therefore, we suggest performing a study of the ontology-based detection of future signals of fertility issues in South Korea that employs a quantitative method involving 2 types of keyword portfolio maps and a qualitative method involving experts.

### Conclusions

A determinants-of-fertility ontology was developed in this study that comprised 6 superclasses, 230 subclasses, and 41 relationships with terminology that comprised 1464 synonyms for the 236 classes. Class concepts of the ontology were included as an EAV model and contained synonyms of the ontology classes such as consumer terms and abbreviations. The ontology can be used to analyze social media data on fertility issues. The DL, content coverage, and structural and representational layers of the ontology were evaluated. The ontology and its terminology were used to detect future signals of fertility issues in South Korea. Our novel determinants-of-fertility ontology provides a framework for collecting and analyzing social media data toward understanding which socioeconomic and cultural factors and fertility policies should be focused on in the future. The analysis of future signals revealed that *violence and abuse* (socioeconomic and cultural factor) and *flexible working arrangement* (fertility policy) were weak signals that might increase rapidly in the future. The findings of this study will help policy makers to develop effective policies for responding to the low fertility rate in South Korea based on examinations of the present and future trends.

## Abbreviations

CQ: competency question
DF: document frequency
DL: description logics
DoD: degree of diffusion
EAV: entity-attribute-value
KIM: keyword issues map
OECD: Organization for Economic Cooperation and Development
TFR: total fertility rate
