# Correction: A Determinants-of-Fertility Ontology for Detecting Future Signals of Fertility Issues From Social Media Data: Development of an Ontology

**DOI:** 10.2196/31601

**Published:** 2021-07-13

**Authors:** Ji-Hyun Lee, Hyeoun-Ae Park, Tae-Min Song

**Affiliations:** 1 Nurse's Office Yeongdeok High School Gyeonggi-do Republic of Korea; 2 College of Nursing and Research Institute of Nursing Science Seoul National University Seoul Republic of Korea; 3 Department of Health Management Samyook University Seoul Republic of Korea

In “A Determinants-of-Fertility Ontology for Detecting Future Signals of Fertility Issues From Social Media Data: Development of an Ontology” (J Med Internet Res 2021;23(6):e25028), the authors noted four errors.

Three errors were in the section “Applying the Ontology to Detect Future Signals” in the *Methods* section of the originally published article.

First, the originally published subheading:

Step 1. Collecting News

has been corrected to:

Step 1. Collecting Data

Second, curly brackets were missing from the following equation for DoD:


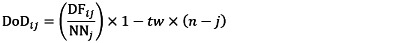


This has been corrected to:


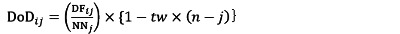


Third, the descriptions of the first and second quadrants were incorrect in the following sentence:

Keywords in the first quadrant, which represent weak signals, have a low average DF but a high average DoD growth rate, and so they may increase rapidly in the future. Keywords in the second quadrant, which represent strong signals, have a trend toward a high average DF and a high average DoD growth rate.

This has been corrected to:

Keywords in the first quadrant, which represent strong signals, have a trend toward a high average DF and a high average DoD growth rate. Keywords in the second quadrant, which represent weak signals, have a low average DF but a high average DoD growth rate, and so they may increase rapidly in the future.

The fourth error was in the section “Applying the Ontology to Detect Future Signals” in the *Results* section of the originally published article.

In the original paper, the words “first” and “second” were switched in the following paragraph:

The weak signals are marked with red rectangle (area A) in the first quadrant of the KIM. The strong signals are marked with blue rectangle (area B) in the second quadrant of the KIM.

This has been corrected to:

The weak signals are marked with red rectangle (area A) in the second quadrant of the KIM. The strong signals are marked with blue rectangle (area B) in the first quadrant of the KIM.

The correction will appear in the online version of the paper on the JMIR Publications website on July 13, 2021, together with the publication of this correction notice. Because this was made after submission to PubMed, PubMed Central, and other full-text repositories, the corrected article has also been resubmitted to those repositories.

